# Impact of early initiation of sodium-glucose cotransporter 2 inhibitor on cardiovascular outcomes in people with diabetes and known or at risk of atherosclerotic cardiovascular disease: Propensity score matched analysis

**DOI:** 10.1371/journal.pone.0277321

**Published:** 2022-11-04

**Authors:** Wen Sun, Alice P. S. Kong, Bryan P. Yan

**Affiliations:** 1 Department of Medicine and Therapeutics, Prince of Wales Hospital, The Chinese University of Hong Kong, Hong Kong, China; 2 Heart & Vascular Institute, The Chinese University of Hong Kong, Hong Kong, China; 3 Li Ka Shing Institute of Health Sciences, The Chinese University of Hong Kong, Hong Kong, China; University of Dundee, UNITED KINGDOM

## Abstract

**Objective:**

We aimed to evaluate the impact of early initiation of sodium-glucose cotransporter 2 inhibitors (SGLT2i) on cardiovascular (CV) outcomes in people with type 2 diabetes (T2D) with known or at risk of atherosclerotic cardiovascular disease (ASCVD).

**Research design and methods:**

T2D with first prescription of SGLT2i (Dx-to-Rx time) ≤12 months were matched with >12 months using propensity score derived from logistic regression. T2D were divided into 3 groups: (i) known ASCVD; (ii) additional CV risk factor(s) and; (iii) without ASCVD or additional CV risk factors. Incidence rates of 3-point major adverse cardiovascular events (MACE, including non-fatal stroke, non-fatal myocardial infarction and CV death) were compared between Dx-to-Rx time ≤12 months and >12 months across 3 subgroups.

**Results:**

Median follow-up was 2.8 years (IQR 2.2 to 3.4). Among 29,309 T2D (mean age 57.6±11.4 years, 59.0% men), 23.6% had established ASCVD and 66.6% had additional CV risk factors. Overall, 19.0% of patients had Dx-to-Rx time ≤12 month which was associated with lower rates of MACE [hazard ratio (HR) = 0.27, 95%CI: 0.17–0.42]. Benefits of early initiation of SGLT2i was observed in patients with additional CV risk factors or known ASCVD but not in those without CV risk factors or ASCVD (P for interaction = 0.001).

**Conclusion:**

Early initiation of SGLT2 inhibitor was associated with lower MACE rates in T2D with known or at risk of ASCVD.

## Introduction

Patients with type 2 diabetes mellitus (T2D) are at risk of adverse cardiovascular events [1,2]. EMPA-REG OUTCOME trial demonstrated in patients with T2D, sodium-glucose cotransporter 2 inhibitor (SGLT2i), Empagliflozin on top of standard care reduced the risk of 3-point major adverse cardiovascular events (MACE) consisting of non-fatal stroke, non-fatal myocardial infarction and cardiovascular death, compared with placebo [3]. The effect was consistent across the spectrum of baseline cardiovascular risk irrespective of prior myocardial infarction (MI) in subgroup analysis [4]. However, in the DECLARE-TIMI 58 trial, dapagliflozin failed to demonstrate lower rate of MACE compared to placebo but did improve cardiovascular mortality or hospitalization for heart failure [5]. In the pre-specified subgroup analysis, dapagliflozin was associated with 16% relative risk reduction in MACE events compared to placebo in patients with history of MI, but no effect in patients without prior MI [6]. Whether patients with T2D with risk factors for but no established atherosclerotic cardiovascular disease (ASCVD) would benefit from SGLT2i remained unclear. The CANVAS trial showed there was no significant reduction in risk of MACE in patients with no ASCVD compared to those with proven ASCVD [7]. Furthermore, optimal timing of initiation of SGLT2i in patients with T2D has not been established. It’s important to determine whether early initiation of SGLT2i might further improve cardiovascular outcomes. Recent evidence suggests that SGLT2i may improve outcomes among survivors of acute MI, particularly when initiated early [8]. In the present study, we retrospectively analyzed consecutive patients with T2D and prescribed SGLT2i in 16 public hospitals in Hong Kong and stratified patients based on known ASCVD or additional CV risk factors (including hypertension and dyslipidemia) aiming to evaluate the impact of early initiation of SGLT2 inhibitor on CV outcomes.

## Methods

### Study design, study population and setting

We performed a retrospective cohort study of consecutive patients with T2D prescribed empagliflozin or dapagliflozin between August 2015 and August 2020 in 16 public tertiary hospitals across Hong Kong identified from the Hospital Authority Clinical Data Analysis & Reporting System (CDARS) which captured all electronic medical records (EMR) of all public hospital admissions (representing >90% of HK population, although private clinic visits are not captured), Accident & Emergency department and out-patient clinic visits (linked to each patient’s unique Hong Kong Identification Number). Data included ICD-9 codes for diagnoses and procedures, prescription records, investigation results and mortality. All patients with exposure to empagliflozin or dapagliflozin were included irrespective of the indications, dosage and frequency of medication. We did not exclude any patients.

### Exposure, outcome measurement, and subgroup definition

The earliest prescription date during study period was considered as the drug initiation date. Using the first diagnosis date of T2D as the index day, we determined the time from diagnosis of T2D to initiation of SGLT2i (Dx-to-Rx time). The whole cohort was divided into two levels of exposure to SGLT2i: Dx-to-Rx time ≤12 months or >12 months. Predictors of early initiation of SGLT2i (i.e., Dx-to-Rx time ≤12 months) were identified through binary logistic regression. As EMR occurring before 2000 was not completely captured by CDARS, missing exact diagnosis date is likely. However, this was not supposed to affect the results, as those diagnoses established before the year of 2000 were regarded to be Dx-to-Rx time >12 months, as defined.

Primary outcome was a composite of 3-point MACE including non-fatal stroke, non-fatal myocardial infarction and cardiovascular death. Secondary outcomes were individual component of MACE. All events of interest were adjudicated independently by 2 clinicians. Observation started with first prescription date of SGLT2i and ended with first occurrence of first ischemic stroke, MI, death, lost to follow-up or study end. The effect of SGLT2i on outcomes were evaluated, adopting intention-to-treat approach, where discontinuation or any interruption of SGLT2i was ignored. Sensitivity analysis was conducted, in which definition of primary outcome was MACE occurring >90 days after last dose of SGLT2i.

Subgroup analyses were conducted to evaluate effect heterogeneity of early initiation across various baseline disease or risk of ASCVD. We divided patients into 3 subgroups: (i) patients with known ASCVD involving coronary artery, peripheral artery or cerebrovascular disease; (ii) CV risk factor(s) other than diabetes and (iii) no known ASCVD or additional CV risk factors.

### Statistical analysis

All diabetic patients with exposure to ≥1 dose of SGLT2i were included in analysis. Patients with Dx-to-Rx time ≤12 months were matched with >12 months using propensity score derived from logistic regression (1:1 with 0.2 caliper), into which all significant multivariate predictors of Dx-to-Rx time ≤12 months (S1 Table in S3 File) were entered. Relative risks of the association between Dx-to-Rx time ≤12 months or >12 months and primary or secondary outcomes were approximated from hazard ratios derived from COX regression adjusted for duration of T2D (time from diagnosis to the end of study), age, gender, baseline lipid lowering, antiplatelet, insulin or oral hypoglycemic therapy to balance the groups after matching, with subgroup by Dx-to-Rx time as interaction term. If proven statistically significant, interaction effect was quantified and reported as excessive incidence rate, assuming the interaction effect was additive. P values for interaction was obtained from test of heterogeneity of Dx-to-Rx time groups differences among subgroups without adjustment for multiple testing. Statistical procedures were conducted using IBM SPSS statistics version 26.

The study was conducted in accordance with the Declaration of Helsinki and the International Conference on Harmonization Guidelines for Good Clinical Practice. The protocol was approved by our institution Clinical Research Ethics Committee (Joint CUHK-NTEC CREC). Written consent was not required for retrospective analysis with non-patient identifiable data.

## Results

### Baseline characteristics of patients before and after propensity score matching

**Fig 1:**
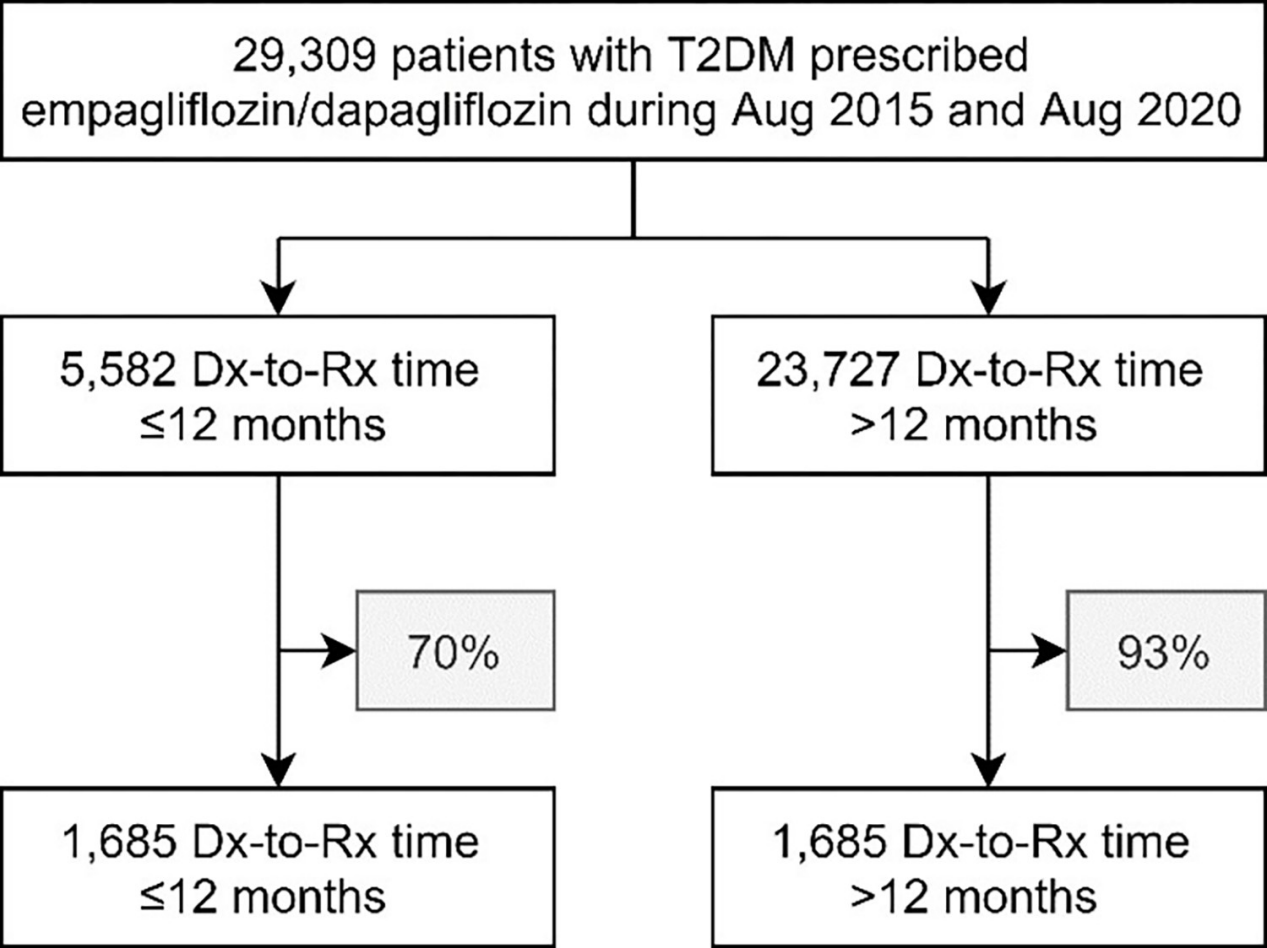
Flow chart for Dx-to-Rx time ≤12 months versus >12 months groups. Proportion of patients not fulfilling propensity matching 1:1 with 0.2 caliper were excluded from subsequent analysis, as shown in the grey boxes.

We identified 29,309 patients (mean age 57.6±11.4 years, female 41.0%) met the pre-defined inclusion criteria (Fig 1), of which 19.0% (n = 5,582/29,309) patients received SGLT2i within 12 months after diagnosis of T2D. Patients initiating SGLT2i early were younger, and less likely to be female and have dyslipidemia, hypertension, concurrent use of insulin, lipid lowering, antiplatelet, or oral hypoglycemic therapy, and more likely to have a shorter duration of T2D (Table 1). Significant predictors for early initiation of SGLT2i were shown in S1 Table in S3 File. The proportions of patients allocated into different subgroups and with Dx-to-Rx time ≤12 month or > 12 months were demonstrated in S1 Fig in S3 File.

**Table 1:** Baseline characteristics in patients with Dx-to-Rx time ≤12 months versus >12 months before propensity score matching.

Characteristics	Dx-to-Rx time ≤12 months (N = 5582)	Dx-to-Rx time >12 months (N = 23727)	P value
Age	55.57±11.01	58.10±11.41	<0.001
Female	2173(38.9%)	9846(41.5%)	<0.001
Dyslipidemia	2505(44.9%)	16464(69.4%)	<0.001
Hypertension	4118(73.8%)	18612(78.4%)	<0.001
Insulin	1624(29.1%)	11406(48.1%)	<0.001
Aspirin	316(5.7%)	3997(16.8%)	<0.001
P2Y12	31(0.6%)	590(2.5%)	<0.001
Acarbose	11(0.2%)	265(1.1%)	<0.001
SU	1591(28.5%)	8019(33.8%)	<0.001
DPP4-inhibitor	277(5.0%)	3399(14.3%)	<0.001
Glitazone	19(0.3%)	626(2.6%)	<0.001
GLP1-agnonist	4(0.1%)	112(0.5%)	<0.001
Statin or ezetimibe	1475(26.4%)	8182(34.5%)	<0.001
Duration of T2D*			
≤3 years	4788(85.8%)	996(4.2%)	<0.001
>3 ≤6 years	794(14.2%)	3958(16.7%)	
>6 ≤9 years	0	5440(22.9%)	
>9 ≤12 years	0	7661(32.3%)	
>12 years	0	5672(23.9%)	

* time from diagnosis of T2D to the end of study.

Totally, 3,370 patients were matched, with well-balanced distribution of baseline characteristics (S2a Table in S3 File). The matched cohort was further stratified into 3 subgroups as defined. The two matched cohorts were stratified by number of CV territory involved as shown in S2b Table in S3 File.

### Risk of major adverse cardiovascular events

Incidence rates of 3-point MACE were compared between Dx-to-Rx time ≤12 months and >12 months across 3 subgroups during a median follow-up of 2.8 years (IQR 2.2 to 3.4). Overall, Dx-to-Rx time ≤12 months was associated with lower rates of MACE (hazard ratio (HR) = 0.27, 95%CI: 0.17–0.42). Subgroup analysis showed similar results in patients with CV risk factors of or known ASCVD but not in patients with neither risk factor nor ASCVD (P for interaction = 0.001, Table 2). Secondary endpoints included three components of MACE. Early initiation of SGLT2i resulted in reduction in risk of MI and CV death, but no significant difference in risk of ischemic stroke (S3a, S3b and S3c Table in S3 File).

**Table 2:** MACE with Dx-to-Rx time ≤12 months versus >12 months in subgroups stratified by presence or absence of known ASCVD or risk factors.

	Dx-to-Rx time ≤12 months			Dx-to-Rx time >12 months			Hazard ratio (95%CI)	P for interaction
MACE	n/N	%	Rate/1000 person-years	n/N	%	Rate/1000 person-years		
All patients	30/1685	1.8	6.0	71/1685	4.2	14.2	0.27 (0.17–0.42)	
Neither ASCVD nor CV risk factor	1/317	0.3	1.1	1/280	0.4	1.3	0.52 (0.03–8.27)	0.001
CV Risk factor only	4/932	0.4	1.4	14/864	1.6	5.3	0.11(0.03–0.42)	
ASCVD	25/436	5.7	20.1	56/541	10.4	35.4	0.49(0.30–0.80)	

**Fig 2:**
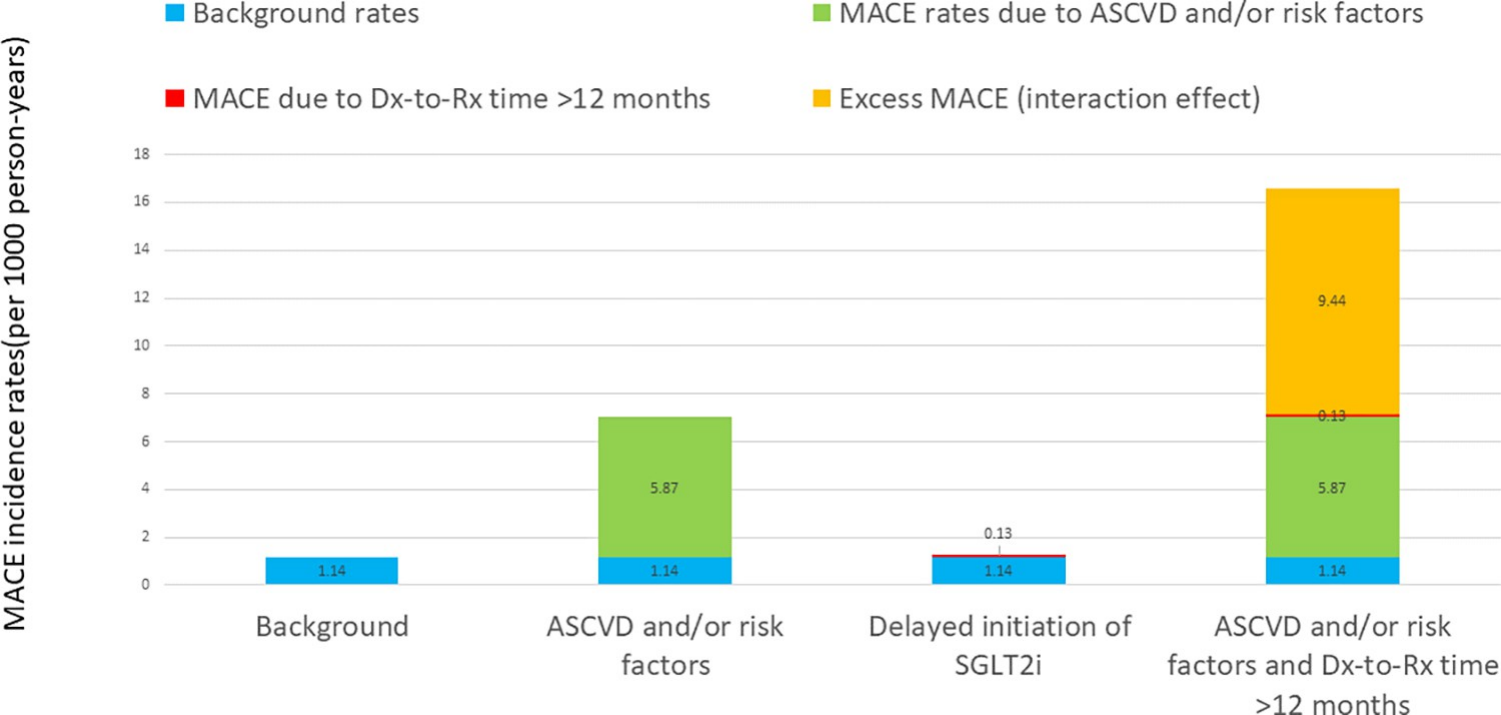
Quantification of interaction effect of ASCVD (and/or risk factors) * delayed initiation of SGLT2i.

To quantify the interaction effect of late prescription of SGLT2i in patients with ASCVD and/or risk factor(s), assuming the excess risk of interaction effect of subgroup by Dx-to-Rx time was additive, we calculated the expected incidence rates for: (i) patients with neither ASCVD nor additional risk factors who had Dx-to-Rx time ≤12 months (as background incidence rate, expected to be lowest); (ii) patients with known ASCVD or CV risk factors who had Dx-to-Rx time ≤12 months (effect of ASCVD or CV risk factors alone); (iii) patients with neither ASCVD nor risk factors who had Dx-to-Rx time > 12 months (effect of delayed initiation of SGLT2i alone); and (iv) patients with known ASCVD or CV risk factors who had Dx-to-Rx time >12 months (combined effect of ASCVD or risk factors and delayed use of SGLT2i with excess risk of interaction effect) (S4 Table in S3 File). ASCVD and/or risk factors alone and delayed use of SGLT2i contributed additional rates of MACE by 5.87 and 0.13 per 1000 person-years, respectively, whereas the excess risk (interaction effect) of a combination of ASCVD/risk factors and Dx-to-Rx time >12 months was quite large (9.44 per 1000 person-years) (Fig 2).

## Discussion

In this study, we demonstrated early initiation of SGLT2i with time from diagnosis of T2D to first prescription of SGLT2i (Dx-to-Rx time) of ≤12 months was associated with lower risk of MACE compared to delayed initiation of SGLT2i. Benefits of early initiation of SGLT2i was observed in patients with additional CV risk factors or known ASCVD but not in those without CV risk factors or ASCVD Our findings highlighted the importance of prompt initiation of SGLT2i to prevent adverse cardiovascular events, in particular, patients who had established ASCVD or with CV risk factors in addition to T2D.

The CV benefits of SGLT2i in patients with known ASCVD appeared robust across clinical trials. The findings of our study filled the gap of evidence of prescribing SGLT2i in patients with cardiovascular risk factor(s) but no proven ASCVD as primary prevention treatment. Moreover, when analyzing individual component of 3-point MACE, we did not detect any statistically significant difference between early and late utilization of SGLT2i with regard to the risk of ischemic stroke, of which the result was consistent with that observed in the EMPA-REG OUTCOME trial [9].

The mechanism of beneficial effect of SGLT2i on cardiovascular outcomes is not fully understood but definitely beyond the improvement in glycemic control [10]. In a single-center study including 16 patients with T2D randomized to dapagliflozin or placebo, myocardial flow reserve (MFR) measured by 13N-ammonia PET/CT was significantly improved in patients on depagliflozin [11]. In another study including 80 patients with T2D, compared with sitagliptin, dapagliflozin decreased potent atherogenic small-dense LDL-C and increased HDL2-C levels after 12 weeks [12]. The DAPA-LVH trial showed dapagliflozin significantly reduced left ventricular mass, body weight, 24-h and nocturnal systolic blood pressure, visceral adipose tissue, subcutaneous adipose tissue, insulin resistance, and high-sensitivity C-reactive protein in patients with T2D [13]. Similarly, the EMPA-HEART CardioLink-6 trial demonstrated mean left ventricular mass indexed to body surface area achieved larger regression than placebo over 6 months, with lowering of overall ambulatory systolic blood pressure and elevation of hematocrit [14]. Improved arterial stiffness and endothelial function were both observed in type 1 diabetic patients prescribed Empagliflozin on top of metformin [15]. A number of mechanisms have been proposed for cardiovascular protective effect of SGLT2i. For diabetic patients, SGLT2i might offer benefit through several molecular and cellular pathways involved in the diabetic-associated ventricular remodelling, including (i) left ventricular hypertrophy; (ii) production of extracellular matrix; (iii) impairment of cardiac metabolism and cardio-myocyte apoptosis; and (iv) increase in cytokines and pro-inflammation [16]. Other hypothesis included that SGLT2i led to changes in epicardial adipose tissue mass [17,18] inhibition of myocardial Na+/H+ exchange, and reduction in preload by osmotic diuresis and afterload by improvement in vascular endothelial function [19–21]. However, questions remained, in that consistent cardiovascular risk reduction was observed in patients without diabetes, which indicated glucose-lowering effect and benefit in cardiovascular outcomes might be dissociated [16]. Optimal timing for the initiation of SGLT2i has not been addressed by either current therapeutic guidelines or other studies. In the sub-analysis from the DECLARE-TIMI 58 trial, SGLT2i was associated with lower risk of MACE in patients with history of MI, but not among those without prior MI, including patients with established ASCVD but no history of MI [6]. By contrast, our studyshowed the benefit might be expanded to patients with ASCVD with or without prior MI, and/or risk factors. It’s noteworthy that the characteristics of cohorts in the two studies were quite different, in that, the cohort in our study were younger, and had a shorter duration of diabetes (i.e., over 95% of patients with duration of ≤6 years, as opposed to 10–11 years in the DECLARE-TIMI 58 trial. Age and the progression of diabetic micro/macrovascular complications might be confounding factors.

## Limitation

Early initiation was defined with an arbitrary cut-off (i.e., ≤12 months). We observed probability of MACE to Dx-to-Rx time on scatter plot, and found a trend that the earlier the timing of first prescription, the lower risk of MACE. Further study is warranted to test whether the correlation is linear or not. It’s worth conducting a subsequent study, to explore the ‘intervention window’, beyond which the benefit would diminish. Second, we did not evaluate the persistence of SGLT2i, and assumed that once patients was put on SGLT2i, there was no discontinuation or interruption occurring during follow-up, adopting intention-to-treat approach. Third, the study cohort was matched for limited factors that would determine the outcomes. Concurrent exposure to other oral hypoglycemic agents was merely roughly matched for numbers of oral medications. Fourth, the cohort was matched using propensity score for duration of diabetes (from first diagnosis of DM to study end), in order that the two groups after matching were comparable, at the expense of loss of sample size. Furthermore, important covariates for patients’ overall risk of worsening heart failure, such as hospitalization for heart failure, bio-marker (e.g. NT-pro BNP) and echocardiogram to assess LV ejection fractions would be informative but which are not routinely performed in public hospitals in Hong Kong.)

## Conclusion

Our study supports current guidelines which recommend SGLT2i for diabetic patients with established ASCVD or at high risk of ASCVD. We demonstrated that early initiation of SGLT2i within 12 months after diagnosis of T2D in patients with known ASCVD or at high risk of ASCVD was associated with better CV outcomes compared to delayed initiation. However, the impact of early initiation was not observed in those with no known ASCVD and no additional CV risk factor.

## Supporting information

S1 File Data of all patients who met inclusion criteria before propensity score matching. (SAV)

S2 File Data of propensity score matched cohort. (SAV)

S3 File Supporting information–contains all the supporting tables and figures. (DOCX)
