# Supplementary material for: Impact of early initiation of sodium-glucose cotransporter 2 inhibitor on cardiovascular outcomes in people with diabetes and known or at risk of atherosclerotic cardiovascular disease: Propensity score matched analysis
Source: PLoS One. 2022 Nov 4;17(11):e0277321. doi: 10.1371/journal.pone.0277321 (PMC9635734; doi:10.1371/journal.pone.0277321)
Supplement: S3 File — (DOCX) [file pone.0277321.s003.docx]

**Supporting Information**


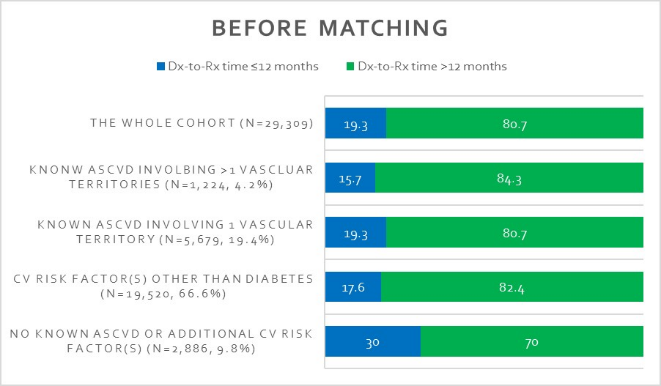


S1 Fig. Proportions of patients with Dx-to-Rx time ≤12 months versus >12 months stratified by subgroups

S1 Table. Predictors for Dx-to-Rx time ≤12 months

| variables | Univariate regression | Multivariate regression |
| --- | --- | --- |
|  | Odds ratio(95%CI) | Odds ratio(95%CI) |
| Age (<40 as reference) |  | |
| ≥40 <50 | 1.04(0.90-1.20) | 1.33(0.14-1.55) |
| ≥50 <60 | 1.69(1.48-1.93) | 2.43(2.12-2.79) |
| ≥60 <70 | 3.00(2.63-3.43) | 4.70(4.09-5.41) |
| ≥70 <80 | 3.66(3.15-4.25) | 6.02(5.13-7.07) |
| ≥80 | 5.17(4.11-6.49) | 7.22(5.66-9.22) |
| Female | 0.90(0.85-0.95) | 0.83(0.78-0.89) |
| Dyslipidemia | 0.36(0.34-0.38) | 0.26(0.24-0.28) |
| Hypertension | 0.77(0.72-0.83) | 0.85(0.79-0.91) |
| Aspirin | 0.30(0.26-0.33) | 0.31(0.27-0.36) |
| P2Y12 | 0.22(0.15-0.32) | 0.40(0.27-0.58) |
| Insulin | 0.24(0.21-0.27) | 0.26(0.23-0.30) |
| Acarbose | 0.18(0.10-0.32) | 0.27(0.14-0.49) |
| SU | 0.78(0.73-0.83) | 0.78(0.71-0.85) |
| DPP4-inhibitor | 0.31(0.28-0.35) | 0.25(0.18-0.33) |
| Metformin | 0.63(0.59-0.67) |  |
| Glitazone | 0.13(0.08-0.20) | 0.31(0.19-0.49) |
| GLP1-agonist | 0.15(0.06-0.41) |  |
| Statin or Ezetimibe | 0.68(0.64-0.73) | 3.22(2.89-3.58) |

S2a Table. Baseline characteristics in patients with Dx-to-Rx time ≤12 months versus >12 months after propensity score matching

| Characteristics | Dx-to-Rx time  ≤12 months  (N=1685) | Dx-to-Rx time  >12 months  (N=1685) | P value |
| --- | --- | --- | --- |
| Dx-to-Rx time |  |  |  |
| Mean ± SD | 4.7 ± 4.0 | 23.6 ± 9.1 |  |
| Median, IQR | 4.1 (0.2-8.2) | 21.6 (16.2-28.9) |  |
| Age | 54.09±11.70 | 55.42±12.11 | 0.001 |
| Female | 625(37.1%) | 642(38.1%) | 0.545 |
| Dyslipidemia | 652(38.7%) | 703(41.7%) | 0.079 |
| Hypertension | 1174(69.7%) | 1199(71.2%) | 0.365 |
| Aspirin | 98(5.8%) | 142(8.4%) | 0.003 |
| P2Y12 | 13(0.8%) | 21(1.2%) | 0.168 |
| Insulin | 616(36.6%) | 586(34.8%) | 0.297 |
| Metformin | 458(27.2%) | 495(29.4%) | 0.157 |
| Acarbose | 6(0.4%) | 4(0.2%) | 0.526 |
| SU | 363(21.5%) | 384(22.8%) | 0.384 |
| Gliptin | 66(3.9%) | 51(3.0%) | 0.158 |
| DPP4-inhibitor | 81(4.8%) | 71(4.2%) | 0.407 |
| Glitazone | 14(0.8%) | 7(0.4%) | 0.125 |
| GLP1-agnonist | 4(0.2%) | 0 | 0.125 |
| Statin or ezetimibe | 352(20.9%) | 344(20.4%) | 0.734 |
| Duration of T2D^†^ |  | | |
| ≤3 years | 922(54.7%) | 889(52.8%) | 0.008^*^ |
| >3 ≤6 years | 763(45.3%) | 788(46.8%) |  |
| >6 years | 0 | 8(0.5%) |  |
| Mean ± SD | 2.95 ± 1.13 | 2.92 ± 1.00 | 0.369 |

^*^ Fisher’s exact test

^†^ time from diagnosis of T2D to the end of study

S2b Table. Baseline characteristics in patients divided by known ASCVD or risk factor(s) in 2 matched cohorts

| Characteristics | Known ASCVD involving >1 territories  (N=205) | Known ASCVD involving 1 territory  (N=772) | CV risk factor(s) other than T2D  (N=1796) | No known ASCVD or additional CV risk factors  (N=597) | P value |
| --- | --- | --- | --- | --- | --- |
| Age | 59.13±10.74 | 59.08±10.12 | 54.64±11.38 | 48.00±12.86 | <0.001^*^ |
| Female | 65(31.7%) | 172(22.3%) | 749(41.7%) | 281(47.1%) | <0.001 |
| Dyslipidemia | 78(38.0%) | 367(47.5%) | 910(50.7%) | 0 | 0.002^†^ |
| Hypertension | 170(82.9%) | 608(78.8%) | 1595(88.8%) | 0 | <0.001^†^ |
| Oral antidiabetics | 123(60.0%) | 517(67.0%) | 1253(69.8%) | 389(65.2%) | 0.012 |
| Insulin | 62(30.2%) | 272(35.2%) | 682(38.0%) | 186(31.2%) | 0.007 |
| Duration of T2D^§^ |  | | | | |
| ≤3 years | 115(56.1%) | 418(54.1%) | 896(49.9%) | 382(64.0%) | <0.001‡ |
| >3 ≤6 years | 90(43.9%) | 350(45.3%) | 896(49.9%) | 215(36.0%) |  |
| >6 years | 0 | 4(0.5%) | 4(0.2%) | 0 |  |
| Dx-to-Rx time  ≤ 12 months | 72(35.1%) | 364(47.2%) | 932(51.9%) | 317(53.1%) | <0.001 |

^*^ Welch one-way ANOVA due to unequal variance (Levene test)

^†^ compared between 3 groups with group of No known ASCVD or additional CV risk factors excluded

^‡^ Fisher’s exact test

^§^ time from diagnosis of T2D to the end of study

S3a Table. MI with Dx-to-Rx time ≤12 months versus >12 months in subgroups stratified by presence or absence of known ASCVD or risk factors.

|  | Dx-to-Rx time  ≤12 months | | | Dx-to-Rx time  >12 months | | | Hazard ratio (95%CI) | P for interaction |
| --- | --- | --- | --- | --- | --- | --- | --- | --- |
| MI | n/N | % | Rate/1000 person-years | n/N | % | Rate/1000 person-years |  |  |
| All  patients | 10/1685 | 0.6 | 2.0 | 27/1685 | 1.6 | 5.5 | 0.33(0.16-0.69) |  |
| Neither ASCVD nor CV risk factor | 0/317 | 0 | 0 | 0/280 | 0 | 0 | NA | 0.992 |
| CV Risk factor only | 3/932 | 0.3 | 1.1 | 5/864 | 0.6 | 1.9 | 0.38(0.08-1.84) |  |
| ASCVD | 7/436 | 1.6 | 5.7 | 22/541 | 4.1 | 13.9 | 0.44(0.18-1.05) |  |

S3b Table. Ischemic stroke with Dx-to-Rx time ≤12 months versus >12 months in subgroups stratified by presence or absence of known ASCVD or risk factors.

|  | Dx-to-Rx time  ≤12 months | | | Dx-to-Rx time  >12 months | | | Hazard ratio (95%CI) | P for interaction |
| --- | --- | --- | --- | --- | --- | --- | --- | --- |
| MI | n/N | % | Rate/1000 person-years | n/N | % | Rate/1000 person-years |  |  |
| All  patients | 14/1685 | 0.8 | 2.8 | 24/1685 | 1.4 | 4.9 | 0.542(0.280-1.051) |  |
| Neither ASCVD nor CV risk factor | 0/317 | 0 | 0 | 0/280 | 0 | 0 | NA | 0.916 |
| CV Risk factor only | 0/932 | 0 | 0 | 0/864 | 0 | 0 | NA |  |
| ASCVD | 14/436 | 3.2 | 11.3 | 24/541 | 4.4 | 15.2 | 0.80(0.41-1.58) |  |

S3c Table. CV death with Dx-to-Rx time ≤12 months versus >12 months in subgroups stratified by presence or absence of known ASCVD or risk factors.

|  | Dx-to-Rx time  ≤12 months | | | Dx-to-Rx time  >12 months | | | Hazard ratio (95%CI) | P for interaction |
| --- | --- | --- | --- | --- | --- | --- | --- | --- |
| MI | n/N | % | Rate/1000 person-years | n/N | % | Rate/1000 person-years |  |  |
| All  patients | 10/1685 | 0.6 | 2.0 | 23/1685 | 1.4 | 4.5 | 0.39(0.18-0.82) |  |
| Neither ASCVD nor CV risk factor | 1/317 | 0.3 | 1.1 | 1/280 | 0.4 | 1.3 | 0.88(0.06-14.13) | 0.124 |
| CV Risk factor only | 2/932 | 0.2 | 0.7 | 9/864 | 1.0 | 3.4 | 0.17(0.04-0.77) |  |
| ASCVD | 7/436 | 1.6 | 5.5 | 13/541 | 2.4 | 8.0 | 0.79(0.31-2.02) |  |

S4 Table. Incidence rates of MACE in patients with delayed initiation of SGLT2i or ASCVD (and/or risk factors) or a combination of both

| Dx-to-Rx time >12 months | ASCVD and/or risk factors | Incidence rate of MACE  (per 1000 person-years) |
| --- | --- | --- |
| Yes | Yes | 16.58 |
| Yes | No | 1.27 |
| No | Yes | 7.01 |
| No | No | 1.14 |
